# Genome-Wide Analysis and Expression Profiling of Glyoxalase Gene Families Under Abiotic Stresses in Cucumber (*Cucumis sativus* L.)

**DOI:** 10.3390/ijms252011294

**Published:** 2024-10-20

**Authors:** Kaili Zhu, Yongxue Zhang, Weiyao Shen, Lishu Yu, Dandan Li, Haoyu Zhang, Chen Miao, Xiaotao Ding, Yuping Jiang

**Affiliations:** 1 College of Ecological Technology and Engineering, Shanghai Institute of Technology, Shanghai 201418, China; z18253926832@163.com (K.Z.); 15921457938@163.com (L.Y.); lddwyzh2022@163.com (D.L.); zhanghy4453542@163.com (H.Z.); 2 Shanghai Key Laboratory of Protected Horticulture Technology, Horticultural Research Institute, Shanghai Academy of Agricultural Science, Shanghai 201403, China; xuezylemon@foxmail.com (Y.Z.); 19542816801@163.com (W.S.); miaochen@saas.sh.cn (C.M.); 3 College of Life Sciences, Shanghai Normal University, Shanghai 200234, China

**Keywords:** cucumber, glyoxalase, abiotic stress, genome-wide identification

## Abstract

The glyoxalase pathway, consisting of glyoxalase I (GLYI) and glyoxalase II (GLYII), is an enzymatic system that converts cytotoxic methylglyoxal to non-toxic S-D-lactoylglutathione. Although the *GLY* gene family has been analyzed in *Arabidopsis*, rice, grape, cabbage, and soybean, cucumber studies are lacking. Here, we analyzed the cucumber *GLY* gene family, identifying 13 *CsGLYI* and 2 *CsGLYII* genes. Furthermore, we investigated the physicochemical properties, phylogenetic relationships, chromosomal localization and colinearity, gene structure, conserved motifs, *cis*-regulatory elements, and protein–protein interaction networks of the CsGLY family. They were primarily localized in the cytoplasm, chloroplasts, and mitochondria, with a minor presence in the nucleus. The classification of *CsGLYI* and *CsGLYII* genes into five classes closely resembled the homologous genes in *Arabidopsis* and soybean. Additionally, hormone-responsive elements dominated the promoter region of *GLY* genes, alongside light- and stress-responsive elements. The predicted interaction proteins of CsGLYIs and CsGLYIIs exerted a significant role in cellular respiration, amino acid synthesis, and metabolism, as well as methylglyoxal catabolism. In addition, the expression profiles of *GLY* genes were distinct in different tissues of cucumber as well as under diverse abiotic stresses. This study is conducive to the further exploration of the functional diversity among glyoxalase genes and the mechanisms of stress responses in cucumber.

## 1. Introduction

Methylglyoxal (MG), also known as pyruvaldehyde, possesses two active groups: an aldehyde group and a ketone group. The α and β-ketoaldehyde with a reactive carbonyl group in the ketone group exhibits strong reactivity, making it the most prevalent active dicarbonyl compound. MG is produced as a byproduct of non-enzymatic reactions through glycolysis and the Calvin cycle, as well as enzymatic pathways of protein and fatty acid metabolism [1]. At low concentrations, it serves as a vital signaling molecule regulating various physiological processes, including cell metabolism, seed germination, plant growth and development, and stress tolerance [2]. However, elevated levels of MG can spontaneously form advanced glycation end-products (AGEs) upon interaction with nucleic acids, proteins, or lipids [2,3], which induce reactive oxygen species (ROS) in cells, while disrupting the stability of the cell membrane, cytoskeleton integrity, and organelle function, as well as reducing the efficiency of enzymatic reactions. To this end, plants have evolved diverse defense mechanisms to detoxify MG and maintain MG balance in plant cells, including glyoxalase systems as well as antioxidant enzymatic and non-enzymatic systems [4].

The glyoxalase system is a highly ubiquitous pathway for the detoxification of MG and is widely distributed in both eukaryotes and prokaryotes [5,6]. Comprising three enzymes, namely, glyoxalase I (GLYI; lactoylglutathione lyase), glyoxalase II (GLYII; hydroxyacylglutathione hydrolase), and glyoxalase III (GLYIII), this system operates through synergistic cascade reactions, along with the independent reaction of GLYIII. Initially, MG is converted to hemithioacetal (HTA) in the presence of reduced glutathione (GSH), followed by its isomerization to S-D-lactose glutathione (SLG) catalyzed by GLYI. Subsequently, SLG is hydrolyzed by GLYII to form D-lactate while recycling the released glutathione back into the system [7]. Apart from the conventional GSH-dependent GLYI-II pathway for MG detoxification, there also exists a shorter GSH-independent MG detoxification pathway involving GLYIII that directly catalyzes the irreversible conversion of MG to D-lactate in a single detoxification step without requiring cofactors [8]. Although the role of GLYIII in stress responses has been revealed, there are limited studies investigating GLYIII in model plants and cereal crops [9,10].

Currently, genome-wide sequencing has facilitated the identification of *GLYI* and *GLYII* genes in model plants and important crops such as *Arabidopsis thaliana* [11], *Oryza sativa* L. [11], *Sorghum bicolor* [12], *Glycine max* [13], *Medicago truncatula* [14], *Cassava* [15], and *Brassica rapa* L. [16]. However, there is limited information available regarding the genome-wide identification of the *GLYIII* gene [17,18]. In terms of classification based on genome-wide analysis, GLYI is categorized as a metalloenzyme and can be further divided into Ni^2+^-dependent and Zn^2+^-dependent types [19]. Three genes in *Arabidopsis* (*AtGLYI2*, *AtGLYI3*, and *AtGLYI6*) and two genes in rice (*OsGLYI8* and *OsGLYI11.2*) have been experimentally shown to be functional and contain all essential binding sites for glyoxalase activity. In contrast, GLYII belongs to the β-lactamase protein family with a binuclear metal center composed of Fe^3+^, Zn^2+^, and Mn^2+^ [20]. The GLYII protein has been identified as a divalent metal cation-binding protein in *Arabidopsis* [21]. However, Ghosh et al. [22] demonstrated that the addition of Zn^2+^ and Fe^2+^ did not activate the activity of GLYII protein in rice; instead, the addition of Co^2+^ was found to be an effective catalyst. Finally, GLYIII belongs to the DJ-1/Pfp1 superfamily (PARK7/*Pyrococcus furiosus* protease I) with a conserved catalytic triad Glu-Cys-His in its active site and requires no cofactors for optimal enzymatic activity [22]. In rice, the conserved cysteine at the N-terminal domain of OsDJ-1C protein can utilize MG as a substrate to produce D-lactate in a GSH-independent manner. Site-directed mutagenesis on this cysteine resulted in the loss of GLYIII activity, confirming the function of cysteine is indispensable for its activity [23]. However, the activity of the GLYIII enzyme is considerably less efficient than GLYI and GLYII enzymes, with only a one-thousandth efficiency compared to them [24]. Consequently, we focus on studying GLYI and GLYII.

The expression and activity of GLYI and GLYII changed significantly under various stress conditions, including hypoxia, salt, drought, heat, cold, and heavy metals [25,26,27]. Additionally, the expression of plant glyoxalase genes was induced by the treatment of hormones and regulators such as abscisic acid (ABA) [28], salicylic acid (SA) [29], and hydrogen peroxide [23]. For example, the transcript and protein levels of GLYI in tomatoes were upregulated in response to salt stress, phytohormones, and osmotic stimulation [28]. In pumpkin seedlings, the expression of *GLYI* was induced by salinity, heavy metals, white light, and MG treatments [30]. The activities of *GLYI* and *GLYII* in onion were induced by low temperature [31], while the expression of *GLYII* in mustard was upregulated by the treatment of salt stress along with heavy metals and ABA [29]. In response to high temperature (HT) stress, rice seedlings exhibited a significant increase in the activities of lipoxygenase, GLYI, and GLYII along with elevated levels of malondialdehyde (MDA), hydrogen peroxide (H_2_O_2_), and proline [32]. The involvement of the glyoxalase system in the regulation of plant abiotic stress responses has been confirmed by omics studies as well as transgenic experiments [33]. Exogenous GSH treatment increased the activities of both GLYI and GLYII but reduced the levels of MG and ROS in mung bean seedlings under HT stress [34]. Similarly, the activities of both GLYI and GLYII were significantly enhanced by an exogenous selenium (Se) treatment in rapeseed (*Brassica napus* L.) seedlings under HT treatment [35]. Overall, the enzyme system is considered a potential biomarker of plant stress tolerance.

Cucumber (*Cucumis sativus* L.), also known as the stinging gourd, is a vital vegetable crop, exhibiting a preference for temperature [36]. The identification and expression analysis of the stress response genes in cucumber at the genome-wide level, together with an in-depth analysis of their molecular mechanisms, can help to improve the stress resistance and cultivate new varieties with superior quality and higher yields. In this study, we identified the members of the GLYI and GLYII families from the Cucumber Genome Database (V2.0) by a bioinformatics analysis and explored the characteristics and potential functions of these family members. A systematic analysis was conducted from various perspectives, including physical and chemical properties, phylogenetic analysis and evolutionary relationships, chromosomal localization and colinearity, genetic structure, conserved motifs, *cis*-regulatory elements, and interacting proteins. Additionally, we also identified proteins and gene expression profiles in different tissues in response to stress, which provide an essential foundation for clarifying the evolutionary levels and functional differentiation among *GLYI* and *GLYII* gene families of cucumber in *Cucurbitaceae* crops.

## 2. Results

### 2.1. Genome-Wide Identification and Phylogenetic Analysis of GLYI and GLYII Proteins in Cucumber

A local Blast search was performed on the reference genome of cucumber using 16 *Arabidopsis* GLYI and GLYII protein sequences as the query objects, and 67 preliminary protein sequences were identified. Proteins containing the glyoxalase domain (PF00903) and having the putative function of lactoylglutathione lyase were classified as CsGLYI proteins, while proteins with the metallo-beta-lactamase domain (PF00753) and the putative function of hydroxyacyl glutathione hydrolase were classified as CsGLYII proteins. Based on the conserved domains, a total of 16 *GLYI* and 12 *GLYII* candidate genes were identified. Subsequently, the NCBI-CDD and Pfam databases were utilized for predicting the domains of each candidate gene. A phylogenetic tree was constructed using MEGA6.0 software, followed by a pairwise alignment analysis to confirm sequence integrity among highly similar sequences. Finally, a total of 13 *CsGLYI* and 2 *CsGLYII* genes were identified with their respective gene locus, chromosome location, open reading frame length, and physicochemical properties, as listed in Table 1.

In regard to the 13 *CsGLYI* genes, the open reading frame (ORF) length of *CsGLYI* members was found to vary considerably, ranging from 426 bp (*CsGLYI9*) to 1089 bp (*CsGLYI5*), with an average length of 708 bp. The theoretical isoelectric points (pI) spanned from 4.66 (*CsGLYI12*) to 9.68 (*CsGLYI7*). The majority of CsGLYI members exhibited acidic pI values, while three proteins (*CsGLYI2*, *CsGLYI6*, and *CsGLYI7*) exhibited obvious alkalinity pI values. Among them, nine *CsGLYIs* were predicted as stable proteins (instability index < 40), while four *CsGLYIs* were predicted as unstable proteins (instability index > 40). Furthermore, the aliphatic index ranged from 72.33 (*CsGLYI2*) to 96.58 (*CsGLYI8*), and the grand average of hydropathicity ranged from −0.685 (*CsGLYI12*) to 0.138 (*CsGLYI8*). Among the 13 *CsGLYI* members, *CsGLYI8* was the sole hydrophobic protein, while the remaining 12 were hydrophilic proteins. CsGLYI proteins were predicted to had multiple subcellular localizations, including in the cytoplasm, mitochondrion, chloroplast, nucleus, plasma membrane, vacuolar, extracellular, endoplasmic reticulum lumen, and cytoplasm region.

Among the *CsGLYII* genes examined in this study, the length of ORF varied from 777 bp for *CsGLYII2* to 987 bp for *CsGLYII1*. The PI values of these proteins ranged from 5.95 (*CsGLYII2*) to 8.74 (*CsGLYII1*). All identified proteins were stable under normal physiological conditions. In addition, the aliphatic index values ranged from 85.70 (*CsGLYII2*) to 87.10 (*CsGLYII1*), indicating a high hydrophobicity for all protein isoforms. Based on the analysis of subcellular localization, it can be postulated that these proteins are mainly localized in mitochondria and chloroplasts.

### 2.2. Phylogenetic Relationship Analysis of Cucumber GLYI and GLYII Genes

**Figure 1:**
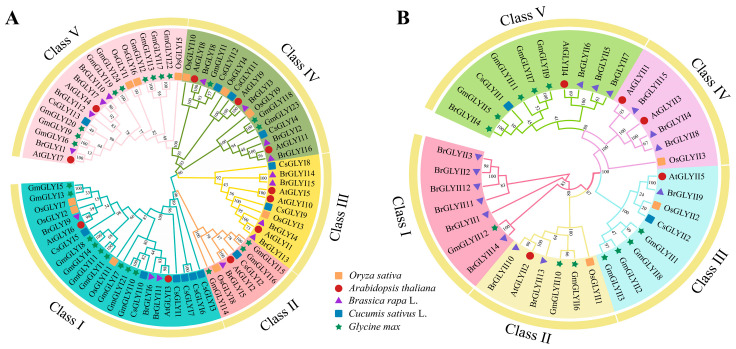
The phylogenetic trees of GLYI (**A**) and GLYII (**B**) from five plant species. A total of 75 GLYI proteins from *A. thaliana* (11), *B. rapa* L. (16), *O. sativa* (11), *G. max* (24), and *C. sativus* (13) were utilized in the phylogenetic analysis of CsGLYΙ (**A**), and 37 GLYII proteins from *A. thaliana* (5), *B. rapa* L. (15), *O. sativa* (3), *G. max* (12), and *C. sativus* (2) were used in phylogenetic analysis of CsGLYΙI (**B**). The proteins from *Arabidopsis*, *B. rapa*, *O. sativa*, *G. max*, and *C. sativus* are indicated by red circles, purple triangles, orange squares, green stars, and blue squares, respectively.

To gain further insight into the phylogenetic relationship between the CsGLYI and CsGLYII families and the GLYI and GLYII proteins in different species, we constructed phylogenetic trees based on 16 sequences from *Arabidopsis*, 31 sequences from *Brassica rapa* L., 41 sequences from *Glycine max*, 14 sequences from *Oryza sativa*, and 13 GLYI sequences and 2 GLYII sequences from cucumber using the neighbor-joining (NJ) method in MEGA 6.0 software. The results indicated that both GLYI and GLYII proteins can be classified into five subfamilies denoted by different colors (Figure 1). Among the GLYIs depicted in Figure 1A, Group I contained the largest number of members, including CsGLYI1, 3, 5, 6, 7, and 10. In contrast, Group II and Group V each contained only one member of the cucumber GLY family (*CsGLYI2* and *CsGLYI13*, respectively), representing the smallest groups. Regarding the GLYII proteins shown in Figure 1B, only Group III and Group V contained CsGLYIIs. Group I comprised six BrGLYIIs and one GmGLYII. Group V showed the greatest diversity of GLYII members with four BrGLYIIs, four GmGLYIIs, one AtGLYII, and one CsGLYII. The aggregation of GLYI and GLYII protein sequences across five species suggests that they may have a similar function or subfunction during species-specific development.

### 2.3. Chromosomal Distribution of Cucumber GLYI and GLYII Genes

**Figure 2:**
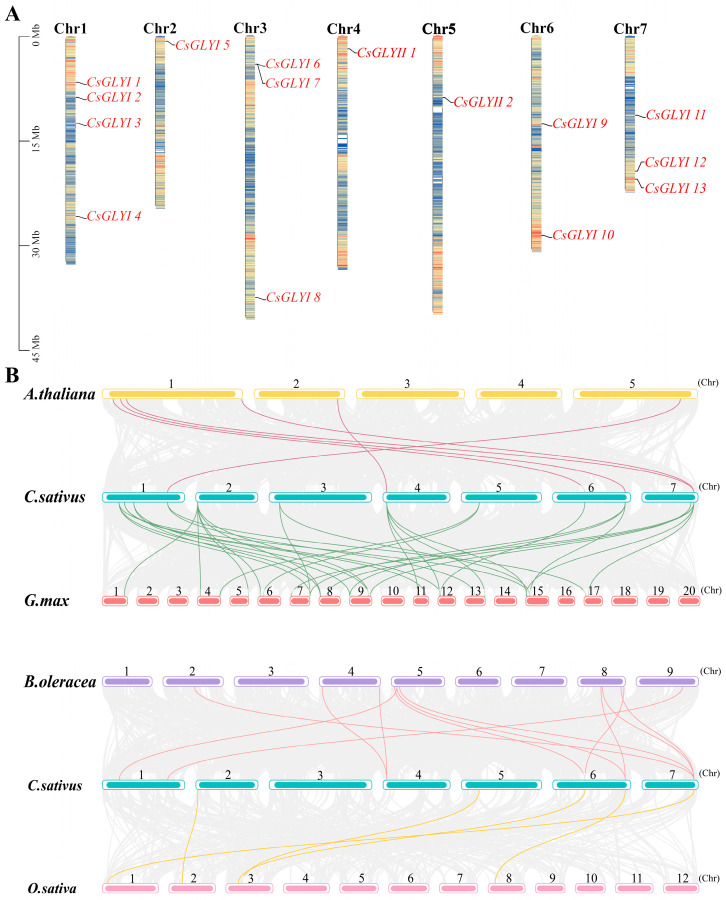
Chromosomal mapping and colinearity analysis of *GLYI* and *GLYII* genes in cucumber. (**A**) Chromosomal localization of *CsGLYI* and *CsGLYII* genes in *C. sativus* chromosomes, with the scale in megabase (Mb). The different colors of the chromosomes represent the gene density. (**B**) Synteny analysis of *GLY* genes between *Arabidopsis thaliana*, *Glycine max*, *Brassica oleracea*, *Oryza sativa*, and *C. sativus*. Duplicated glyoxalase genes are connected by colored lines between the two relevant chromosomes. The number represent the chromosome. Gray lines in the background indicated the collinear blocks within cucumber and other plant genomes, while the lines highlighted the syntenic GLY gene pairs.

The results of chromosomal positioning revealed that 13 *CsGLYI* and 2 *CsGLYII* genes were unevenly anchored on the seven chromosomes (Figure 2A). The largest distribution of *CsGLYI* genes was found on Chromosome (Chr) 1, with four identified (*CsGLYI1*, *2*, *3*, and *4*). Chr 3 and Chr7 each contained three *CsGLYI* genes: *CsGLYI6*, *7*, *8* and *CsGLYI11*, *12*, *13*, respectively. Two *CsGLYI* genes were located on Chr 6, while only one *CsGLYI* gene was present on Chr 2. As for the two *CsGLYII* genes, *CsGLYII1* was located on Chr 4 and *CsGLYII2* on Chr 5. No *CsGLYII* genes were found on the remaining chromosomes.

A synteny analysis was conducted to investigate the potential evolution of the *GLY* gene family in cucumber, using the *GLYI* and *GLYII* genes from *C*. *sativus* L., *A. thaliana*, *B*. *oleracea*, *G*. *max*, and *O*. *sativa* (Figure 2B). Among the *GLYI* genes, it was found that cucumber had a colinear relationship with 5, 26, 9, and 4 genes discovered in the genomes of *A. thaliana*, soybean, *B*. *oleracea*, and rice, respectively. Among these colinear gene pairs, *CsGLYI5* had the largest number of homologous genes in soybean (*GmGLYI1*, *3*, *4*, *5*, *8*, and *11*). *CsGLYI13* contained four homologous genes in both soybean and *B. oleracea* (*GmGLYI6*, *9*, *20*, *24* and *BolGLYI01*, *06*, *07*, *10*), indicating that *CsGLYI5* and *GLYI13* were conserved in the CsGLYI family.

In the *GLYII* gene family, a consistent pattern of colinearity was observed between one *CsGLYII* gene and one *AtGLYII* gene, between two *CsGLYII* genes and six *GmGLYII* genes, between one *CsGLYII* gene and two *BolGLYII* genes, and between one *CsGLYII* gene and one *OsGLYII* gene. Among these colinear gene pairs, *CsGLYII1* had the most orthologous genes in soybean with four genes (*GmGLYII4*, *5*, *7*, and *9*), while *CsGLYII2*, in turn, had two homologous genes (*GmGLYII2* and *GmGLYII3*). These findings indicated that the evolutionary profile of *CsGLY* genes in cucumber is relatively conserved, suggesting their potential origin from orthologous counterparts in other plant species.

### 2.4. The Conserved Domain and Gene Structure of Cucumber GLYI and GLYII Genes

**Figure 3:**
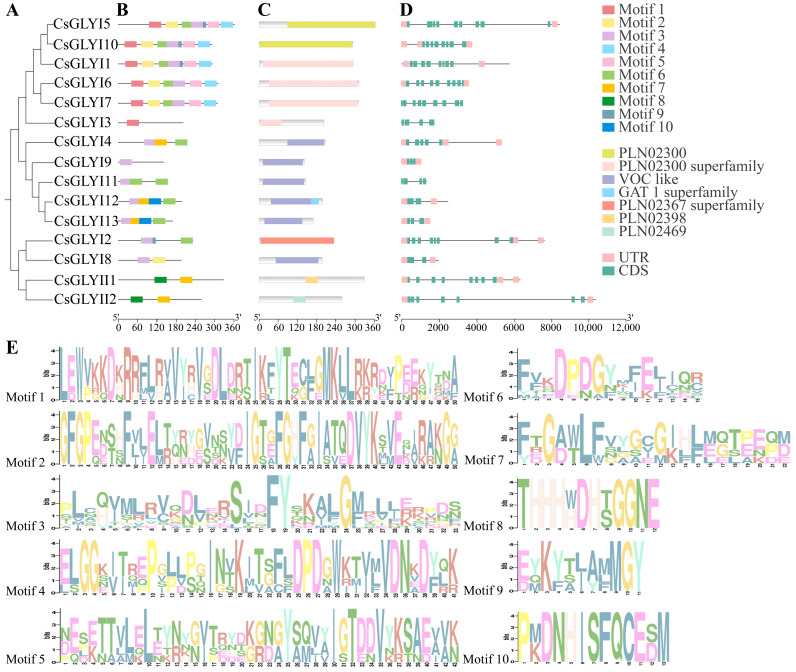
Phylogenetic tree, motif analysis, and gene structure of cucumber *GLYI* and *GLYII* genes. (**A**) An unrooted tree of the full-length amino acid sequences of the 13 CsGLYI and 2 CsGLYII proteins was generated using the neighbor-joining method with 1000 bootstrap replicates using MEGA 6.0 software. (**B**,**C**,**E**) The distribution of the CsGLYI and CsGLYII conserved motifs was analyzed by MEME with 10 as the maximum number of motifs. Each specific motif is marked by a different colored box. (**D**) All the exons are shown in filled green boxes and the introns are indicated by black lines. The 5′-UTR regions and the 3′-UTR regions are shown using pink boxes, which also indicate the direction of the gene.

The conserved domains, gene structures, and exon/intron structural patterns of the *CsGLYI* and *CsGLYII* genes were investigated based on their phylogenetic relationships (Figure 3A). All 13 members of the CsGLYI family were found to contain the glyoxalase domain, and two members of the CsGLYII family contained the metallo-β-lactamase domain. The results demonstrated that the majority of CsGLYI and CsGLYII proteins within the same group exhibited a consistent pattern of motif distribution in each group (Figure 3B). For instance, most CsGLYI proteins contained Motif 3 other than CsGLYI3, and except for CsGLYI3, 8, and 9, Motif 6 was identified in all CsGLYI proteins. As for the cucumber GLYII family, Motifs 7 and 8 were present in both CsGLYII proteins, suggesting that they are indispensable for fulfilling the functions of the CsGLYIIs. CsGLYI proteins possess PLN02300, PLN02367, and vicinal oxygen chelate (VOC) super family domains that are visible in the GLYI enzymes of other species (Figure 3C). Among the two GLYII members, CsGLYII1 contained PLN02398 domains and CsGLYII2 contained PLN02469 domains, indicating that they possess the domain signature of hydroxyacylglutathione hydrolase (PLN02398 and PLN02469 super family). The *CsGLYI* and *CsGLYII* genes exhibit similar number of exons and introns, but there are significant differences in their arrangement and length (Figure 3D). Among the *CsGLYI* genes, *CsGLYI5*, *6*, and *7* had the highest number of exons with nine each, whereas *CsGLYI8* and *CsGLYI12* contained only two exons each. However, *CsGLYII* genes showed variation in their number of exons and introns, with *CsGLYII1* containing the most.

### 2.5. Analysis of Cis-Regulatory Element Prediction in CsGLYI and CsGLYII Promoters

**Figure 4:**
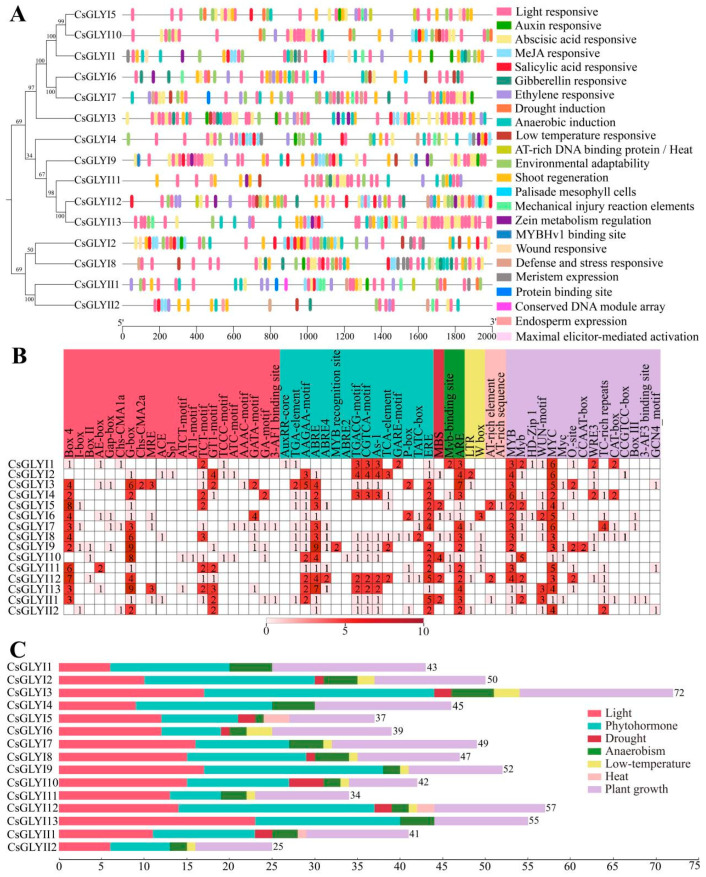
Analysis of *cis*-regulatory elements of *CsGLYI* and *CsGLYII* genes. (**A**) *Cis*-regulatory elements in the promoters of 13 *CsGLYI* and 2 *CsGLYII* genes. Different elements are presented by various color symbols, and their position in the figure indicates their relative position on the promoter. (**B**) The relative proportions of different *cis*-regulatory elements in the promoters of *CsGLYI* and *CsGLYII* genes are indicated in the chart. *Cis*-regulatory elements sharing identical or similar functions are represented by the same color. (**C**) The number of various *cis*-regulatory elements in the promoters of each *CsGLYI* and *CsGLYII* gene in cucumber.

To further investigate the mechanisms by which *CsGLYI* and *CsGLYII* genes are regulated by plant hormones and defense stress, a comprehensive analysis was conducted on the 2000 bp upstream promoter sequence of each gene (Figure 4). A total of 60 predicted regulatory elements were identified, among which eukaryotic promoter elements such as CAAT-box and TATA-box had the highest number. Additionally, numerous other elements associated with plant hormones, light responses, defense and stress responses, MYB-binding sites, meristem expression, endosperm expression, and plant growth and development were identified. Among them, there were many elements related to plant hormones, light response, and plant growth and development. Those related to hormone responses included auxin response elements (AuxRR core and TGA element), ABA response elements (AAGAA motif, ABRE, ABRE2, ABRE4, and MYB recognition site), MeJA response elements (TGACG motif and CGTCA motif), and SA response elements (SARE). Those related to plant growth and development included environmental adaptation (MYB and MYB), palisade mesophyll cells (HD Zip 1), mechanical injury response (WUN motif), defense and stress response (TC rich repetitive sequences), bud regeneration (MYC and MYC), regulation of zein metabolism (O_2_ site), and wound response (WRE3). However, most types were light-responsive elements (21 kinds). In the cucumber *GLYI* gene family, the promoters of *CsGLYI3* and *CsGLYI4* contained the highest number of *cis*-regulatory elements, accounting for 11.30% and 9.42%, respectively. Conversely, the *CsGLYI11* contained the least number of components, accounting for only 5.34%. Among the cucumber *GLYII* gene family, *CsGLYII1* exhibited a higher number of regulatory elements compared to *CsGLYII2*.

### 2.6. Interaction Network of CsGLYI and CsGLYII Proteins

**Figure 5:**
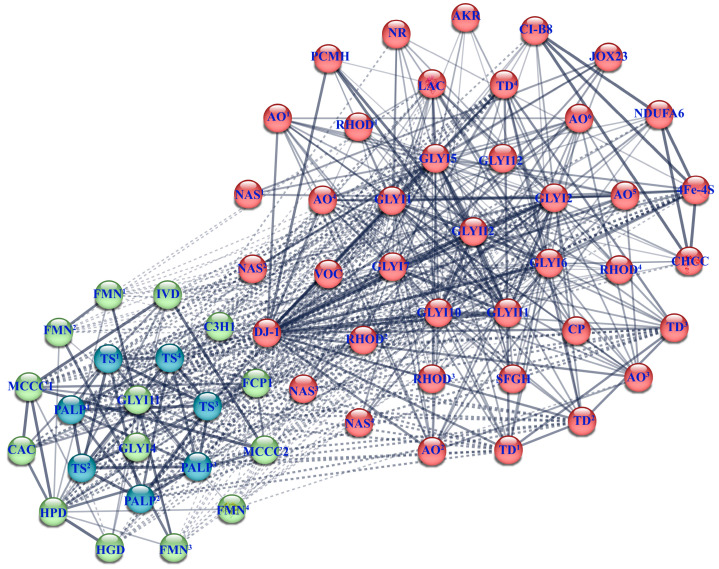
Prediction of protein–protein interaction (PPI) networks of CsGLYI, CsGLYII, and their related proteins using the STRING tool. The different nodes represent the interacting proteins, and the full names and abbreviations of the proteins in the figure are as follows: amine oxidase: AO; aldo_ket_red domain-containing protein: AKR; biotin carboxylase: CAC; C3H1-type domain-containing protein: C3H1; cysteine protease: CP; DJ-1/PfpI domain-containing protein: DJ-1; FMN hydroxy acid dehydrogenase domain-containing protein: FMN; FAD-binding PCMH-type domain-containing protein: PCMH; FCP1 homology domain-containing protein: FCP1; 4Fe-4S ferredoxin-type domain-containing protein: 4Fe-4S; 4-hydroxyphenylpyruvate dioxygenase: HPD; homogentisate 1,2-dioxygenase: HGD; isovaleryl-CoA dehydrogenase: IVD; 23 kDa jasmonate-induced protein-like: JOX23; L51_S25_CI-B8 domain-containing protein: CI-B8; methylcrotonoyl-CoA carboxylase: MCCC; NADH dehydrogenase [ubiquinone] 1 alpha subcomplex subunit 6: NDUFA6; nicotianamine synthase: NAS; nitroreductase domain-containing protein: NR; PALP domain-containing protein: PALP; rhodanese domain-containing protein: RHOD; S-formylglutathione hydrolase: SFGH; threonine dehydratase: TD; tryptophan synthase: TS; zf-CHCC domain-containing protein: CHCC.

To investigate the regulatory functions of the CsGLYIs and CsGLYIIs, we constructed an interaction network consisting of 26 cucumber proteins using the STRING program (http://string-db.org, accessed on 27 May 2024). Among these proteins, nine CsGLYI and two CsGLYII proteins were identified to interact with the other 25 proteins in cucumber. As shown in Figure 5, the protein–protein interaction (PPI) network contained 64 nodes and 495 edges, indicating extensive connections among these proteins and their involvement in various biological processes. Several CsGLYI and CsGLYII proteins, including CsGLYI1, 2, 5, 6, 7, CsGLYII1, and CsGLYII2 interacted with oxidative enzymes associated with cellular redox regulation and lactate catabolic metabolism. In rice, the interaction between OsGLYI-11.2 (Os08g09250) and a zinc finger protein OsDHSRP1 (Os02g05692) was identified by a yeast two-hybrid (Y2H) system, which provided important information regarding plant adaptation and regulation under abiotic stresses [37]. In the protein interaction network of this study, GLY protein and a zinc finger protein CHCC (Csa_2G432800) of cucumber also interacted with each other and participated in the mitochondrial electron transport pathway and responded to adversity stress by regulating intracellular signaling molecules, suggesting that they may play an important role in regulating plant abiotic stress responses.

### 2.7. Tissue-Specific Gene Expression Analysis of CsGLYIs and CsGLYIIs

**Figure 6:**
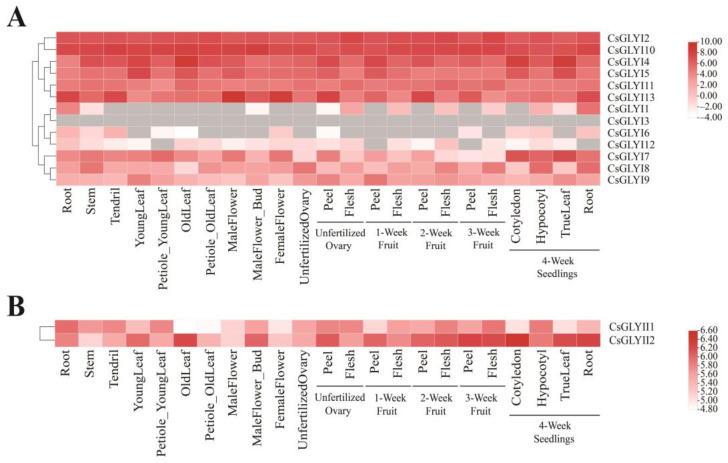
Expression pattern of cucumber *GLYI* (**A**) and *GLYII* (**B**) genes. The transcriptional levels of *CsGLYI* and *CsGLYII* genes in 23 tissues of cucumber were investigated based on public transcriptome data, and the genome-wide expression of *CsGLYI* and *CsGLYII* genes was shown on heatmaps using FPKM values. Gery: Gene not expressed.

To investigate the tissue specificity of *GLY* genes in cucumber, a heatmap of 13 *CsGLYI* and 2 *CsGLYII* gene expression profiles in 23 different tissues during vegetative and reproductive development was generated (Figure 6). For example, *CsGLYI1* showed exclusive expression in the root, stem, male flower bud, peel of unfertilized ovary, flesh of unfertilized ovary, flesh of 1-week fruit, flesh of 2-week fruit, flesh of 3-week fruit, hypocotyl of 4-week seedlings, true leaf of 4-week seedlings, and root of 4-week seedlings. *CsGLYI3* was not expressed in any of the tissues or organs examined. With the exception of *CsGLYI1*, *3*, *5*, *6*, and *12*, the remaining nine members of the *CsGLYI* gene family showed ubiquitous expression in all 23 cucumber tissues (Figure 6A). Additionally, *CsGLYI2*, *4*, *5*, *10*, *11*, and *13* showed a relatively higher expression in all 23 organs and tissues. Among the *CsGLYII* gene family, *CsGLYII1* and *CsGLYII2* were expressed in 23 different tissues, while *CsGLYII1* exhibited a high expression specifically in the root of 4-week seedlings (Figure 6B). These findings suggested that the tissue-specific expression patterns of the different members of the *CsGLY* gene family are diverse.

### 2.8. Expression of CsGLYIs and CsGLYIIs in Response to Abiotic Stresses and Hormone Treatments

**Figure 7:**
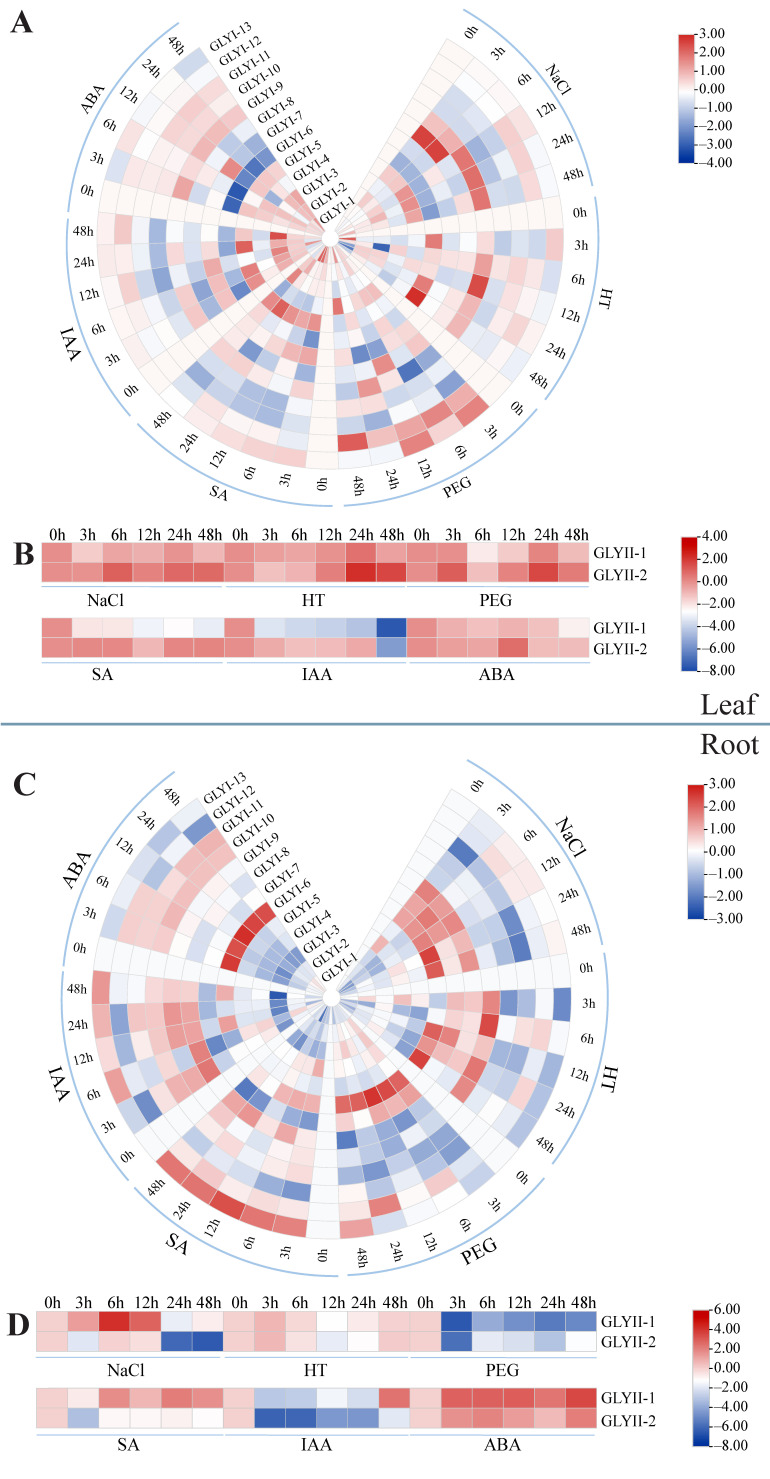
Expression profiles of *CsGLYI* and *CsGLYII* genes in different tissues in response to various abiotic stress and hormone treatments. The transcriptional levels of *CsGLYI* and *CsGLYII* genes in leaves (**A**,**B**) and roots (**C**,**D**) were investigated based on transcriptome data. Significant changes compared to CK have been calculated using a Student’s *t*-test, and the detailed information is included in Supplementary File S3 Table S1.

We evaluated the relative expression levels of 15 *CsGLY* genes under various abiotic stresses and hormone treatments, including NaCl, PEG, HT, ABA, indole-3-acetic acid (IAA), and SA, using quantitative reverse transcription polymerase chain reaction (qRT-PCR) analysis. As shown in Figure 7, the expression of *CsGLYs* in response to different treatments was distinct. The results showed that for two different tissues of roots and leaves, in the *GLYI* gene family, under NaCl treatment, the expression of *GLYI2*, *3*, *5*, *9*, and *12* was increased in leaves, but *GLYI6* and *GLYI10* were both in a downward trend; the expression of *GLYI4*, *6*, *7*, and *GLYI8* in roots was upregulated, while *GLYI1*, *2*, *3*, and *11* showed a downregulated trend. After treatment with PEG, it was observed that the expression level of *GLYI12* was significantly elevated in leaves; at the same time, *GLYI1*, *5*, *8*, *9*, and *10* were downregulated in roots, and the expression of *GLYI6* in roots was increased. Under HT stress conditions, *GLYI13* was consistently suppressed in roots. The results showed that under the three abiotic stresses, the expression levels of *GLYI1*, *2*, *5*, *8*, *9*, *12*, and *13* in leaves were higher, while the expression levels of *GLYI4*, *7*, *10*, and *11* were lower; the expression levels of *GLYI6* and *GLYI7* were higher in roots, while the expression of *GLYI1*, *2*, *3*, *5*, *11*, and *13* was consistently lower. The expressions of *GLYI1*, *2*, and *3* in the NaCl treatment were opposite in leaves and roots. These results indicate that the expression of *GLYI* gene members is tissue-specific in response to abiotic stress. In response to various hormonal stresses, *GLYI5* exhibited an increased expression in leaves, while the expressions of *GLYI4*, *6*, *8*, *10*, *11*, and *12* were found to be downregulated; the relative expression of *GLYI4* and *GLYI13* was significantly elevated in roots, while *GLYI1*, *3*, *7*, and *11* exhibited a consistent downregulation. Upon subjecting the cucumber to IAA, the expression levels of *GLYI8* and *GLYI9* were the highest in roots. Under the ABA treatment, the expression level of *GLYI6* was the highest in roots and the lowest in leaves. The results showed that under the hormonal treatment, the expression levels of *GLYI2*, *3*, and *5* were increased in leaves, while the expression levels of the remaining GLYI genes exhibited a decrease; the expressions of *GLYI6*, *8*, *9*, and *10* were found to be upregulated in roots, whereas the expressions of *GLYI1*, *2*, *3*, *5*, *7*, and *12* were observed to be downregulated.

In the *GLYII* gene families, in response to abiotic stress (NaCl, HT, and PEG), there was a decrease in the expression levels of *GLYII1* in both root and leaf tissues. Conversely, the expression of *GLYII2* was significantly higher in leaves compared to roots. Under the PEG treatment, the expression levels of *GLYII1* and *GLYII2* in roots were significantly decreased. The expression level of *GLYII1* in roots was significantly higher than that in leaves under the SA and ABA treatments. Conversely, the expression levels of *GLYII1* in leaves and *GLYII2* in roots and leaves were downregulated under the IAA treatment. Notably, after 48 h of ABA treatment, the expression levels of *GLYII1* and *GLYII2* both reached their peak in roots. The expression patterns of the various glyoxalase members are different, indicating the various glyoxalase members play different roles in distinct stress pathways.

## 3. Discussion

Methylglyoxal (MG) is the most prevalent active dicarbonyl compound and a cytotoxic metabol. The glyoxalase pathway is the main mechanism for the detoxifying of MG and plays a key role in various plant physiological processes, including stress response [38], seed germination [39], plant aging [27], nutrient regulation [40], signal transduction [41], starch synthesis [42], and pollen development [43], which suggests that the glyoxalase pathway enhances plant adaptability to multiple stresses [2]. In this study, a total of 13 *CsGLYI*s and 2 *CsGLYII*s were identified in cucumber. The *CsGLYI* and *CsGLYII* gene families can each be divided into five subgroups, similar to those identified by Sun et al. [44]. Genes within the same subfamily may share similar functions; therefore, we found that the more similar the motif compositions, the greater the similarity among *CsGLY* sequences, further validating the reliability of our classification approach for *CsGLY* genes. In the *CsGLYI* gene family, each subfamily possesses a homologous *AtGLYII* gene (Figure 1A). In the *CsGLYII* gene family, some branches exclusively consist of *GLY* members from *Brassica napus*. L. and soybean (Figure 1B), such as *BrGLYII1*, *2*, *3*, *11*, *12*, *14*, and *GmGLYII12*. These findings indicated that these two families have undergone asynchronous evolution in the genomes of *Brassica napus* L., soybean, and *Arabidopsis*.

Two types of divalent metal-dependent ions have been identified in GLYI proteins: Zn^2+^ and non-Zn^2+^ (mainly Ni^2+^). Previous studies have demonstrated that the requirement for specific divalent ions varies between prokaryotes and eukaryotes. For instance, Zn^2+^ is essential for the optimal activity of GLYI in humans and yeast, while those in *E. coli* rely on Ni^2+^ [45]. In plants, two types of ion-dependent GLYIs have been identified. For instance, in *Arabidopsis*, AtGLYI2 is dependent on Zn^2+^, and an extended amino acid sequence is present in AtGLYI2, which is characteristic of Zn^2+^-dependent GLYI, and the deletion of this additional amino acid sequence in AtGLYI3 and AtGLYI6 is Zn^2+^-independent, as evidenced by the fact that Zn^2+^-activated GLYI proteins generally have a longer amino acid length than Ni^2+^-activated GLYI proteins [11,21]. Therefore, analysis of the length and sequence of a GLYI protein can predict its metal-dependent specificity. In this study, CsGLYI2 and AtGLYI2 proteins were clustered in the same group (Figure 1A), indicating a possible Zn^2+^dependence. Furthermore, CsGLYI5 and CsGLYI10 clustered with the Ni^2+^-dependent members in *Arabidopsis* (Figure 1B), indicating their classification as Ni^2+^-dependent CsGLYI proteins. The protein sequence alignment of cucumber GLYI enzymes with GLYI enzymes of other species (Supplementary File S1: Figure S1) revealed that there were some additional amino acid sequences in the CsGLYI2 protein, which belonged to the Zn^2+^-dependent proteins, while the other 12 CsGLYI proteins lacked these additional amino acids, which belonged to the Zn^2+^-independent proteins. Both types of GLYI proteins contain conserved metal-binding residues (H/QEH/QE) [11,46]. Multiple sequence alignment results showed (Supplementary File S1: Figure S1) that in GLYI proteins, CsGLYI1, CsGLYI2, CsGLYI3, CsGLYI5, CsGLYI6, CsGLYI7, and CsGLYI10 have a conserved glyoxalase domain (PF00903) (Figure 3C), which has conserved metal-binding sites (H/QEH/QE) at the N-terminus. The crystal structures of GLYII proteins from humans and *Arabidopsis* indicate that GLYII proteins belong to the metal-β-lactamase superfamily and require divalent cations for their activity [7,47,48]. The major metal center of AtGLYII5 is reported to be Fe(III)Zn(II) [47]. AtGLYII2 has been shown to bind mixtures of Zn, Fe, or Mn and also shows positive synergism in metal binding [49]. In this study, both CsGLYII proteins contain a metal-binding motif (THHHXDH) and an active site motif (C/GHT) (Supplementary File S2: Figure S2), and both CsGLYII proteins contain a metal-binding motif (THHHXDH) and an active site motif (C/GHT). It may also have functional GLYII enzyme activity, similar to GLYII proteins in *Arabidopsis* and rice, but the dependence of GLY genes on metal ions has yet to be demonstrated experimentally.

The majority of CsGLYI proteins were predominantly localized in the cytoplasm, followed by chloroplasts, mitochondria, and the nucleus (Table 1). The CsGLYII proteins were mainly localized in mitochondria and chloroplasts (Table 1). Chloroplasts and mitochondria are the organelles responsible for photosynthesis and respiration, respectively, and their metabolic activity is considered a significant source of ROS in MG production [50]. Under stress conditions, the components of chloroplasts and mitochondrial undergo glycosylation, while excessive MG inhibits photosynthesis and disrupts mitochondrial function [51]. For example, sorghum contains the SbGlyI8/8.1 protein that potentially possesses a nuclear localization signal, facilitating the conversion of nuclear MG to SLG [12]. OsGLYI8 is localized within the nucleus where it effectively mitigates DNA damage induced by MG. This also suggests that glyoxalase in cucumber may also play a vital role in protecting the photosynthetic system and maintaining mitochondrial function.

It has been proven that the expression of *GLY* genes is specific to different tissues and developmental stages [13,14,16,18]. The expression patterns of *GLY* genes in cucumber exhibit tissue-specific and stress-responsive variations, as revealed by our analysis of publicly available expression data and RT-qPCR (Figure 7). For example, in *Populus*, *PtGLYI1* and *PtGLYI14* were highly expressed in the stem xylem, while *PtGLYII5* was mainly expressed in leaves [52]. In grapes, *VvGLYI1* displayed the highest expression level in leaves, while *VvGLYI2* was highly expressed in both leaves and tendrils. *VvGLYI3* showed a high expression specifically in tendrils and *VvGLYI4* was predominantly expressed in the ovules of young shoots at 20 days after anthesis [18]. In Chinese cabbage, *BrGLYI6* and *BrGLYI11* exhibited high expression levels in corner fruit, whereas *BrGLYI9* showed a strong expression specifically in corner fruit walls; *BrGLYI5* and *BrGLYI2* were highly expressed in roots, while *BrGLYI8* and *BrGLYI11* were abundantly expressed in flower buds. Additionally, callus tissue displayed high expressions of *BrGLYI1*, *7*, *10*, *14*, and *15* [16]. The *GmGLYI* and *GmGLYII* genes in soybean exhibited high expression levels in both below-ground and aboveground tissues, while *GmGLYI* genes such as *GmGLYI1*, *11*, and *22* exhibited moderate expression in seed tissues but showed low or no expression in other sites [13]. In the gene expression profile data, the expression levels of gly genes except *CsGLYI3* were increased in roots. The expression levels of CsGLYI genes except *CsGLYI3* and *CsGLYI12* were significantly increased in the roots of 4-week seedlings. The expression of *CsGLYI1*, *3*, *6*, and *12* in the different tissues and organs of cucumber was low. The expression levels of *GLYII2* were highest in the peel of 3-week fruit. The expression of the *CsGLY* gene varied across tissues, suggesting a potential impact on tissue development.

Concurrently, a significant correlation exists between GLY enzymes and plant abiotic stress tolerance. In rice, exposure to various treatments, including salinity, desiccation, temperature extremes, ABA, and SA, has been demonstrated to induce increased *OsGLYII* expression [27]. The responses of different members of the *GmGLYI* and *GmGLYII* families in soybean to salt, drought, and ABA treatments exhibited considerable variation. Specifically, *GmGLYII9* and *GmGLYII10* exhibited strong downregulation under all three treatment conditions, while significant upregulation was observed for *GmGLYII16* [13]. In *Brassica campestris*, *BnaGLYI27* displayed high upregulation under both heat and drought stresses [53]. In maize leaf blades, the relative expression patterns of *ZmGLYI1* and *ZmGLYI3* exhibited a tendency to increase initially followed by a decrease with the extension of treatment time under drought, salt, and HT treatments. Conversely, *ZmGLYI2* exhibited an increasing trend followed by a decreasing trend across all stress treatments [54]. Our findings indicate that the *CsGLYI* and *CsGLYII* gene family members exhibited differential responses to abiotic stresses such as HT, salt, drought, and hormones (ABA, IAA, SA). Notably, following the 150 mM NaCl treatment, multiple *GLYI* genes showed a significant increasing trend. Moreover, during HT treatment, the majority of *CsGLYI* gene expression levels were highly upregulated, and the highest expression levels were observed when subjecting the plants to a heat stress condition of 35 °C for 12 h. Following hormone treatment, the expression levels of the CsGLYI genes were significantly low, and *GLYII2* showed a higher expression level than *GLYII1.* Each member exhibited a distinct expression pattern in response to a specific stress treatment.

## 4. Materials and Methods

### 4.1. Identification and Basic Information of GLYI and GLYII Genes in Cucumber

Sequences of the members of the *GLYI* and *GLYII* gene families were downloaded from the Cucurbit Genomics Database v 2.0 (http://cucurbitgenomics.org/v2/, accessed on 19 October 2023) website. *Arabidopsis* GLYI and GLYII protein sequences were obtained from TAIR 10 (http://www.arabidopsis.org, accessed on 5 February 2024). The corresponding putative sequences of cucumber were identified using these query sequences through the BLAST program (v 2.14.0). Furthermore, the Hidden Markov Model (HMM) files for the conserved domains of the GLYI and GLYII proteins, including the lactoylglutathione lyase domain (PF00903) and the metallo-beta-lactamase domain (PF00753), were queried in the Pfam database v 37.0 (http://pfam.xfam.org/, accessed on 12 November 2023) to identify candidate *GLYI* and *GLYII* genes in the cucumber genome. The expected E-value cutoff was set at 1 × 10^−5^, and HMMER v 3.0 software was employed for the search. Subsequently, redundant sequences were excluded to retain a single, unique candidate *CsGLYI* and *CsGLYII* gene. The physicochemical properties of cucumber GLYI and GLYII proteins were analyzed using TBtools software (v 2.119). The subcellular localization of proteins was predicted by Cell-PLoc v 2.0 (http://www.csbio.sjtu.edu.cn/bioinf/Cell-PLoc-2, accessed on 14 November 2023), CELLO v 2.5 (http://cello.life.nctu.edu.tw/, accessed on 14 November 2023), and WoLF PSORT v 2.0 (http://www.genscript.com/wolf-psort.html, accessed on 14 November 2023).

### 4.2. Phylogenetic Analysis of the Cucumber GLYI and GLYII Gene Family

The *GLYI* and *GLYII* protein sequences from cucumber, *A. thaliana*, *B. rapa* L., *G. max*, and *O. sativa* L. species were aligned using the ClustalX program with default settings. Phylogenetic trees were constructed using the neighbor-joining method in MEGA v 6.0, with the following construction parameters: bootstrap value of 1000 and pairwise deletion. Subsequently, these trees underwent further refinement on the EvolView online platform v 2.0 (https://evolgenius.info//evolview-v2, accessed on 17 November 2023).

### 4.3. Chromosome Localization and Colinearity Analysis

To gain a more intuitive understanding of the distribution of *GLYI* and *GLYII* genes in *C. sativus* chromosomes, the location information of *GLYI* and *GLYII* genes on chromosomes was obtained by downloading the Chinese Long v3. GFF3 file from the Cucumber Genome Database v 2.0 (http://cucurbitgenomics.org/v2/, accessed on 19 October 2023) and using TBtools software v 2.119 to extract chromosome density information from the genome annotation files.

### 4.4. Analysis of Conserved Motifs, Gene Structure, and Function Domain

The exons, introns, and untranslated regions of each cucumber *GLYI* and *GLYII* gene were retrieved from the GFF3 file and analyzed using the online tool GSDS v 2.0 (http://gsds.gao-lab.org/, accessed on 25 November 2023). The conservative nucleotide sequences in cucumber GLYI and GLYII proteins were predicted using the MEME website v 5.5.7 (http://memesuite.org/, accessed on 25 November 2023) with default parameters except for the maximum number of motifs set to 10. Pfam database (v 37.0) analysis was performed on all predicted cucumber GLYI and GLYII proteins to identify domain positions (http://pfam.xfam.org, accessed on 25 November 2023).

### 4.5. Analysis of Cis-Regulatory Elements

The promoter region 2000 bp upstream of the start codon for all *CsGLYI* and *CsGLYII* gene members was extracted from the cucumber genome sequences using TBtools software. Subsequently, these sequences were then submitted to the PlantCARE database v 1.0 (https://bioinformatics.psb.ugent.be/webtools/plantcare/html/, accessed on 26 December 2023) for the identification of *cis*-regulatory elements.

### 4.6. Analysis of the Protein–Protein Interaction Network

The protein sequences of 13 *CsGLYI* and 2 *CsGLYII* genes were submitted to the STRING online database v 12.0 (http://string-db.org, accessed on 27 May 2024) to determine specific protein interactions between *GLYI* and *GLYII* genes and other cucumber-related proteins. The parameters were set as follows: Organism—*Cucumis sativus* L.; Maximum number of interactors—no more than 50; Minimum required interaction score—medium confidence (0.400); Number of clusters—5; K-means clustering method employed; Line thickness indicates data support strength; Disconnected nodes are hidden in the network.

### 4.7. Gene Expression Profile Analysis

To investigate the expression profiles of cucumber *GLYI* and *GLYII* genes across various tissues and organs, the transcriptome data of *CsGLYI* and *CsGLYII* genes were retrieved from the bioproject PRJNA312872 of the CuGenDB database v 2.0 (http://cucurbitgenomics.org/v2/, accessed on 5 June 2024). RNA-seq data were obtained from the following tissues: root, stem, tendril, young leaf, petiole of young leaf, old leaf, petiole of old leaf, male flower, male flower of bud, female flower, unfertilized ovary, peel of unfertilized ovary, flesh of unfertilized ovary, peel of 1-week fruit, and flesh of 1-week fruit. The expression profiles of the cucumber *GLYI* and *GLYII* genes in each sample were subjected to a clustering analysis followed by the generation of a heatmap using TBtools software v 2.119.

### 4.8. Plant Materials and Treatment

The cucumber cultivar “Chunqiu Wang No. 3” was selected as the plant material for this experiment, which was conducted within the glass greenhouse of the Chongming National Engineering and Technology Research Center for Institutional Agriculture, Shanghai Academy of Agricultural Sciences (Shanghai, China). Seeds selected for fullness and orderliness were soaked in warm water at 55 °C for 20 min and then transferred to room temperature water at 28 °C for 4 h before being left to germinate in a dark chamber at 28 °C for 1 day, followed by sowing in a greenhouse equipped with seedling substrate in a 50-hole dish. The greenhouse was maintained at a constant day temperature of 25 °C and night temperature of 18 °C, with a relative humidity of 80% throughout the experimental period. Once the seedlings reached the one-true-leaf stage, their roots were carefully washed and transplanted into foam plates embedded with small holes (36.5 × 26 × 2 cm^3^) for hydroponics. Subsequently, the roots were immersed in a dark plastic basin (42 × 30 × 15 cm^3^) containing nutrient solution and placed inside an oxygen pump to ensure a continuous oxygen supply for 24 h. When the seedlings had grown three leaves, they were selected for the experimental treatment.

At the three-true-leaf stage, the cucumber seedlings were subjected to various treatments including NaCl (150 mmol/L), 5% polyethylene glycol (PEG) 6000, high temperature (35 °C), IAA (1 mmol/L), SA (5 mmol/L), and ABA (100 μmmol/L). The NaCl and PEG 6000 were added to a nutrient solution, while hormones were applied through leaf spraying. To provide a high-temperature treatment for cucumber roots at 35 °C, a heating rod was placed in each pot. The leaves and roots from plants subjected to different treatments were harvested at 0, 3, 6, 12, 24, and 48 h post-treatment. Each experiment was replicated at least three times. All samples were rapidly frozen in liquid nitrogen and stored at −80 °C for subsequent RNA isolation.

### 4.9. RNA Extraction and qRT-PCR Analysis

A TaKaRa Mini BEST Universal RNA Extraction Kit (Takara Biomedical Technology Co., Beijing, China) was utilized for total RNA extraction from cucumber leaves and roots in accordance with the manufacturer’s instructions. The quality and concentration of each RNA sample were determined using a NanoDrop ND-spectrophotometer (NanoDrop Technologies, Wilmington, DE, USA). Reverse transcription of RNA (1 µg treated with DNase I) into cDNA was performed using a Prime Script RT reagent kit (Takara, Beijing, China). The cucumber β-actin gene (ID number: CsaV3_2G011610) was employed as the internal control to normalize the expression levels of the target genes across different samples. Three biological replicates and three technical replicates were employed.

## 5. Conclusions

In this study, we conducted a comprehensive study of the cucumber glyoxalase gene family (*CsGLYI* and *CsGLYII*) through a genome-wide sequence analysis. Our findings unveiled the presence of 13 *CsGLYI* and 2 *CsGLYII* genes. Phylogenetic analysis, conserved motif prediction, and gene expression patterns have provided insights into the potential functions of the glyoxalase gene family in plant development and response to abiotic stresses. Notably, members of *CsGLYI* and *CsGLYII* families exhibited distinct responses to various abiotic stresses, including high temperatures, salinity, drought, and hormone treatments. These responses were manifested by variable expression levels observed in both roots and leaves. The bioinformatics and expression pattern analyses of the cucumber glyoxalase gene family in this study can facilitate the identification of candidate genes for cloning and further functional characterization, thereby providing a theoretical basis for investigating the biological functions of glyoxalase genes and their applications in cucumber breeding.

## Figures and Tables

**Table 1:** List of identified *GLYI* and *GLYII* genes in cucumber (*Cucumis sativus* L.) along with their detailed information and localization.

Gene Name	Gene Locus	Chromosome Location	ORF Length (bp)	No. of AAs	MW (kDa)	Isoelectric Point (pI)	Instability Index	Aliphatic Index	GRAVY	Protein Subcellular Localization
*CsGLYI1*	CsaV3_1G010700	chr1:6,646,583–6,652,442(+)	885	294	33.41	5.19	27.88	87.48	−0.370	Cyt
*CsGLYI2*	CsaV3_1G013530	chr1:8,811,864–8,819,621(+)	699	232	25.96	8.51	39.13	72.33	−0.405	Cyt, Mit, Chl
*CsGLYI3*	CsaV3_1G020720	chr1:12,512,239–12,514,044(+)	609	202	22.81	6.18	54.85	85.84	−0.491	Cyt, Nuc, Chl, Mit
*CsGLYI4*	CsaV3_1G040650	chr1:25,868,017–25,873,475(−)	621	206	22.75	7.12	35.85	87.09	−0.401	Cyt, Mit, Chl
*CsGLYI5*	CsaV3_2G002080	chr2:758,978– 767,569(+)	1089	362	40.30	6.86	33.04	79.17	−0.296	Cyt, Chl
*CsGLYI6*	CsaV3_3G004980	chr3:4,148,612–4,152,269(−)	936	311	35.46	9.13	28.09	85.21	−0.359	Cyt, PlM, Mit, Vac, Ext, Nuc, Enr
*CsGLYI7*	CsaV3_3G005000	chr3:4,161,990–4,165,350(+)	930	309	35.11	9.68	25.71	92.39	−0.284	Cyt, Mit, Chl, Vac, Enr
*CsGLYI8*	CsaV3_3G046080	chr3:37,657,505–37,659,528(−)	591	196	21.04	5.14	38.49	96.58	0.138	Cyt, Mit, Chl
*CsGLYI9*	CsaV3_6G017010	chr6:12,691,165–12,692,257(−)	426	141	15.80	5.32	34.37	79.43	−0.491	Cyt, Chl
*CsGLYI10*	CsaV3_6G048970	chr6:28,666,313–28,670,159(+)	879	292	32.66	5.23	25.53	82.77	−0.347	Cyt, Nuc
*CsGLYI11*	CsaV3_7G022460	chr7:11,385,869–11,387,256(+)	438	145	16.23	5.94	51.61	80.07	−0.282	Cyt, Mit, Chl, Nuc
*CsGLYI12*	CsaV3_7G030670	chr7:19,411,864–19,414,390(+)	597	198	22.72	4.66	54.31	74.80	−0.685	Cyt, Nuc
*CsGLYI13*	CsaV3_7G032540	chr7:20,551,756–20,553,316(+)	510	169	19.25	5.67	59.14	91.07	−0.262	Cyt, Chl, Nuc
*CsGLYII1*	CsaV3_4G002450	chr4:1,484,192–1,490,622(+)	987	328	36.18	8.74	37.25	87.10	−0.195	Mit, Chl
*CsGLYII2*	CsaV3_5G011130	chr5:7,090,413–7,100,929(+)	777	258	28.70	5.95	31.56	85.70	−0.400	Cyt, Mit, Chl

bp: base pair; No. of AAs: number of amino acids; MW: molecular weight, kDa: kilodalton; pI: isoelectric point; Cyt: cytoplasm; Mit: mitochondrion; GRAVY: grand average of hydropathicity; Chl: chloroplast; Nuc: nucleus; PlM: plasma membrane; Vac: vacuolar; Ext: extracellular; Enr: endoplasmic reticulum lumen. The “+” and “−” represent the negative and positive strands in the direction of the gene, respectively.

## Data Availability

Data are contained within the article and Supplementary Materials.

## Supplementary Materials

The supporting information can be downloaded at https://www.mdpi.com/article/10.3390/ijms252011294/s1.
