# Supplementary material for: Genome-Wide Analysis and Expression Profiling of Glyoxalase Gene Families Under Abiotic Stresses in Cucumber (Cucumis sativus L.)
Source: Int J Mol Sci. 2024 Oct 20;25(20):11294. doi: 10.3390/ijms252011294 (PMC11508195; doi:10.3390/ijms252011294)
Supplement: Supplementary file 1 [file ijms-25-11294-s001.zip › Supplementary File S1 Figure S1 Multiple Sequence alignments of GLYI domains.pdf]

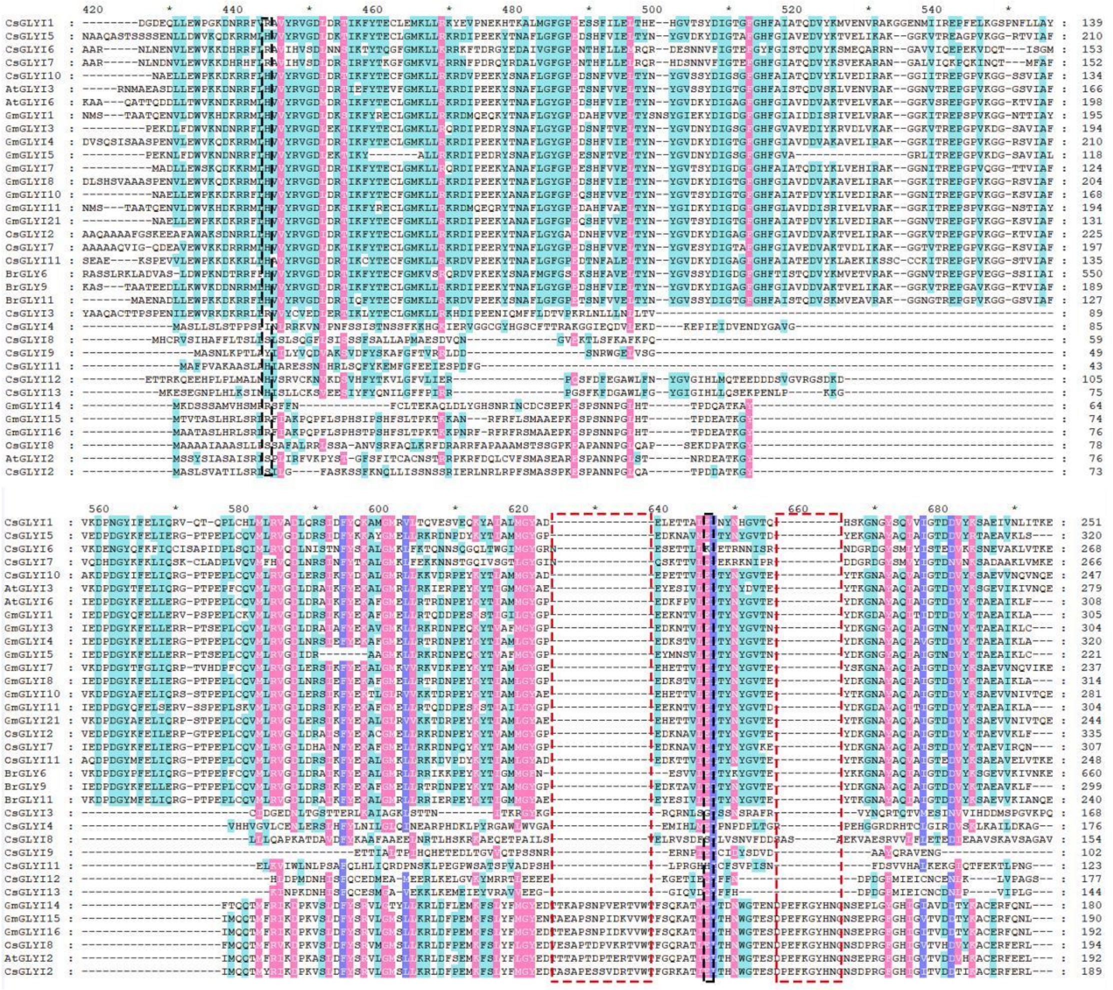

Additional file 1: Figure S1. Multiple Sequence alignments of GLYI domains. N-terminal GLYI domains of GLYI proteins of *A. thaliana*, *B. rapa.* L., *O. sativa*, *G. max* and *C. sativus*.L. were aligned using MEGA6 and edited using the GeneDoc program. The conserved residues (H/E/H/E) for metal binding are shown with black boxes and specific regions for Zn^{2+}-dependence are in red boxes.
