# Supplementary material for: Genome-Wide Analysis and Expression Profiling of Glyoxalase Gene Families Under Abiotic Stresses in Cucumber (Cucumis sativus L.)
Source: Int J Mol Sci. 2024 Oct 20;25(20):11294. doi: 10.3390/ijms252011294 (PMC11508195; doi:10.3390/ijms252011294)
Supplement: Supplementary file 1 [file ijms-25-11294-s001.zip › Supplementary File S2 Figure S2 Multiple Sequence alignments of GLYII domains.pdf]

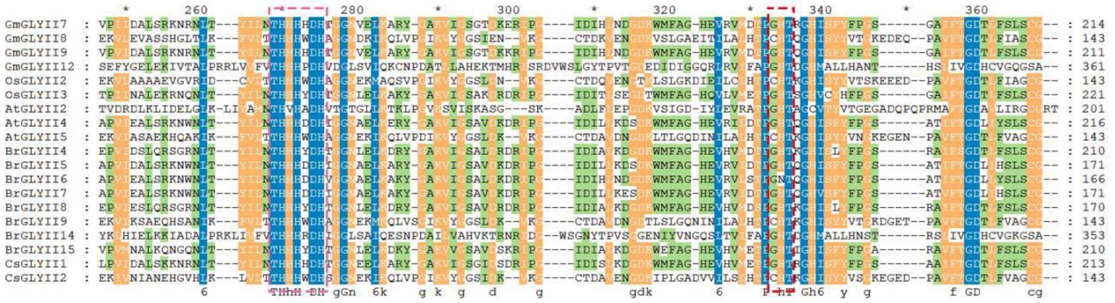

Additional file 2: Figure S2. Sequence alignments of full length of GLYII proteins. Multiple sequence alignments were conducted with full length protein sequences along with GlyII proteins of *A. thaliana*, *B. rapa* L., *O. sativa*, *G. max* and *C. sativus* L. The conserved active motif G/CHT are indicated in red boxes. The conserved metal binding sites are marked with pink boxes.
